# A two dimensional semi-continuum model to explain wetting front instability in porous media

**DOI:** 10.1038/s41598-021-82317-x

**Published:** 2021-02-05

**Authors:** Jakub Kmec, Tomáš Fürst, Rostislav Vodák, Miloslav Šír

**Affiliations:** 1 Joint Laboratory of Optics, Faculty of Science, Palacký University in Olomouc, Olomouc, 771 46 Czech Republic; 2 Dpt. Mathematical Analysis and Applications of Mathematics, Faculty of Science, Palacký University in Olomouc, Olomouc, 771 46 Czech Republic; 3 South Bohemian Research Center of Aquaculture and Biodiversity of Hydrocenoses, Faculty of Fisheries and Protection of Waters, University of South Bohemia, České Budějovice, 370 05 Czech Republic

**Keywords:** Hydrology, Mathematics and computing

## Abstract

Modelling fluid flow in an unsaturated porous medium is a complex problem with many practical applications. There is enough experimental and theoretical evidence that the standard continuum mechanics based modelling approach is unable to capture many important features of porous media flow. In this paper, a two-dimensional semi-continuum model is presented that combines ideas from continuum mechanics with invasion percolation models. The medium is divided into blocks of finite size that retain the nature of a porous medium. Each block is characterized by its porosity, permeability, and a retention curve. The saturation and pressure of the fluids are assumed to be uniform throughout each block. It is demonstrated that the resulting semi-continuum model is able to reproduce (1) gravity induced preferential flow with a spatially rich system of rivulets (fingers) characterized by saturation overshoot, (2) diffusion-like flow with a monotonic saturation profile, (3) the transition between the two. The model helps to explain the formation of the preferential pathways and their persistence and structure (the core and fringe of the fingers), the effect of the initial saturation of the matrix, and the saturation overshoot phenomenon.

## Introduction

The flow of liquids through porous media is a long standing, well-researched, and yet still not fully understood problem. There are two broad areas that draw heavily on understanding porous media flow—oil recovery^1^, and vadose zone hydrology^2,3^. In oil recovery applications, two immiscible and incompressible fluids—water and oil—move in a complex space of pores in a reservoir rock. In vadose zone hydrology, the two fluids are water (incompressible and wetting) and air (compressible and non-wetting). Many other applications of porous media flow have appeared, such as carbon sequestration^4^, the transport of radionuclides in the soil^5^, filtration^6^, hydrogen storage^7^, geothermal engineering^8^, and applications in biological systems and biotechnology^9^. This paper concentrates on the flow of water through soil which is not fully saturated. The lack of understanding of vadose zone hydrology is one of the main bottlenecks in our understanding of the hydrological cycle^10^.

Problems concerning fluid flow are notoriously difficult because of the non-linearity caused by inertial forces (what is called the convective term in the Navier–Stokes equations). In the porous media setting, the flow velocity is usually so small that inertial forces can be neglected. Still, the problem is difficult because the pressure in the wetting fluid at each point is determined by the interplay between hydrostatic forces caused by gravity and capillary forces caused by the presence of the menisci of air–water interfaces, which are in turn determined by the geometry of the porous matrix. Since the geometry of the matrix is so complex that it cannot be fully resolved in any reasonable model, some simplification or approximation is needed.

Traditionally, porous media flow was modeled in the continuum mechanics framework^11^. The mathematical tools of continuum mechanics (infinitesimal calculus and partial differential equations) were standard and ready to be used for the description of porous media flow. Continuum mechanics uses the concept of the reference volume element to transition from naturally discontinuous pore-level quantities to continuous and differentiable macroscopic fields (e.g. pressure, saturation). Various forms of mass conservation equations for these fields are then derived in the form of partial differential equations, the most famous of which is the Richards’ equation (RE)^12^.

Over the years, there has been a growing body of experimental evidence of observed unsaturated porous media flow regimes that were not easy to describe or explain by the standard continuum mechanics based approach^2,3^. The most notorious discrepancy between theory and observation has been what is called the finger flow regime, in which an initially dry porous medium is exposed to a uniform and constant influx of water at the upper boundary. This phenomenon has been studied extensively since the early 1970s^13,14^. While the RE predicts a stable waterfront that moves as a travelling wave downward through the porous matrix, experiments often show the evolution of a spatially rich system of rivulets (fingers) that conduct water downwards through preferential pathways, leaving a substantial part of the matrix almost dry^15–19^. The tips of the fingers are usually almost fully saturated while the tail leaves an under-saturated trace. This phenomenon is called the saturation overshoot. The fingers are stable in the sense that subsequent infiltration proceeds through these pre-wetted preferential paths^15^. It has been proved that the RE is fundamentally incompatible with finger flow because it does not admit saturation overshoot^20–22^. There is enough experimental evidence of the dependence of the finger velocity, width, and overshoot magnitude on the initial saturation and flux magnitude^23–27^. Several extensions of the RE have been proposed^28–37^; however, none is able to reproduce all these observed dependencies of the finger like regime^2^.

Since the 1970s, a different modelling approach has been evolving, which is based on percolation theory and cellular automata^38,39^. In this framework, the porous matrix is modelled by a discrete grid of pores which are either empty or full, and the local discrete rules of pore filling and emptying are executed on such a grid. Various flavours of this approach have been introduced under the names of percolation, invasion percolation, modified invasion percolation, etc.^40–45^ Although some features of unsaturated porous media flow may be captured very well by these models (especially the rich spatial structure of the preferential pathways), the discrete nature of the models makes it hard to recover the saturation overshoot behaviour. Moreover, discrete models usually do not capture time in a physically meaningful way. The local percolation rules usually get the order of the pore-filling (or draining) events right, however, the physical times of the events cannot be recovered. Zhao et al.^46^ reported an unprecedented comparison of 14 state-of-the-art pore-scale models to reproduce recent experimental observations of multiphase fluid flow in porous media.

The seminal work of Glass et al.^47^ tried to combine the virtues of both the continuum and discrete models. The authors proposed an approach called Macro Modified Invasion Percolation in which the porous matrix is divided into macroscopic (not infinitesimal) blocks that retain the characteristics of a porous medium. Each block is fully characterized by its wetting pressure (i.e. the pressure needed for water to invade and percolate the block and make it conductive) and its draining pressure (i.e. the pressure needed for air to displace water from the block). The authors use the model successfully to simulate three different settings—finger flow^48,49^, the buoyancy-driven migration of gas in a water saturated sand column^49,50^, and water flow through a rough-walled fracture^51^.

Inspired by this success, Kmec et al.^52^ proposed a semi-continuum model (the term was coined by DiCarlo^53^), which treats space in a discrete manner but keeps time continuous. It was demonstrated that a one-dimensional version of this semi-continuum model is able to successfully reproduce qualitatively and quantitatively all the features of one dimensional saturation overshoot behaviour reported in the literature.

In this paper, we present a two-dimensional version of the model which is a straightforward extension of the 1D case^52^. The model is again based only on well-established physics, measurable parameters, and material characteristics. We show that the model is able to reproduce both the finger-like regime (for small initial saturation of the matrix) and the diffusion-like regime (for large initial saturation), and the transition between the two. In the finger-like regime, the model reproduces the persistence of the fingers and captures their spatial heterogeneity well. Further, the model helps to explain the inner structure of the finger—the mobile core and immobile fringe—that is observed in experiments. The well-known two-dimensional experiments of Glass et al.^15,47,54–56^ and Rezanezhad et al.^57^ are reproduced by the model. The proposed model is not a numerical scheme to solve the RE and does not reduce to the RE even in the limit of vanishing block size.

## Methods

Kmec et al.^52^ proposed a one-dimensional version of a semi-continuum model of unsaturated porous media flow. The purpose of the model in 1D was to replicate experiments in a narrow sand-filled tube while keeping the model as simple as possible. Here we extend the model to two spatial dimensions because in 2D there are many more features of the finger flow that can be tested. A 2D model aims to capture experiments in a vertical Hele-Shaw cell filled with sand used for experiments e.g. by DiCarlo^25^. The distance between the two parallel plates is small and thus the dependence of the quantities of interest on this coordinate can be neglected; hence the notion of 2D porous media flow.

The porous medium is modelled as a regular rectangular grid of $N \times M$ small square blocks of uniform size $dx \times dx$. The blocks are not considered to be infinitesimal and the limit $dx \rightarrow 0$ will not be performed (see the “Discussion” section). Each block of the material is assumed to retain the characteristics of a porous medium and so each block is fully characterized by two material functions—the pressure-saturation dependence in the wetting phase (known as the retention curve) and the dependence of hydraulic conductivity on saturation. The amount of the wetting fluid (water in our case) in each block is captured by the moisture content (saturation) and the pressure. Both these quantities are assumed to be uniform inside each block but continuously changing with time. For simplicity, the non-wetting phase (air in our case) is assumed to have zero pressure everywhere.

Each block is denoted by its row and column indices [*i*, *j*]. The model simulates the motion of the wetting fluid inside the 2D porous medium by tracking the following three quantities:

- the saturation $S_t(i,j)$ [-] of the wetting phase in each block at time *t*. Saturation is assumed to be uniform throughout each block but varying continuously in time;
- the pressure $P_t(i,j)$ [Pa] of the wetting phase in each block at time *t*. Pressure is assumed to be uniform throughout each block but varying continuously in time;
- the fluxes $q_t[(i_1,j_1) \rightarrow (i_2,j_2)]$ [${\text {ms}}^{-1}$] of the wetting phase between blocks $(i_1,j_1)$ and $(i_2,j_2)$ at time *t*. The fluxes are assumed to be continuous in time.

The evolution of these three quantities is modeled by the following three rules.

### Saturation update

The update of saturation in each block is based on simple mass balance.

$$\theta \partial _t S(t,x) + \text{ div }( q(t,x) ) = 0,$$

where $\theta$ [-] denotes the porosity of the material. This mass balance is implemented by the following discrete scheme

1 $$\begin{aligned}&\frac{\theta }{dt} \left[ S_{t+dt}(i,j)- S_t(i,j) \right] \nonumber \\&\quad =\frac{1}{dx} \left[ q_t[(i-1,j) \rightarrow (i,j)] - q_t[(i,j) \rightarrow (i+1,j)] + q_t[(i,j-1) \rightarrow (i,j)]- q_t[(i,j) \rightarrow (i,j+1)] \right) . \end{aligned}$$

### Pressure update

Next, the pressure in each block is updated according to the retention curve. Here we assume that all the blocks share the same retention curve. See the “Discussion” section for more considerations regarding this assumption. The standard retention curve consists of the main wetting and draining branches, which are both assumed to be non-decreasing^58^. It is well known that the retention curve exhibits substantial hysteresis, i.e. the pressure-saturation relation also depends on the history of the system. The main wetting and draining branches are modelled by the standard van Genuchten relation^58^;

2 $$\begin{aligned} P = \frac{1}{\alpha } \Big ( \big (S^\frac{-1}{m}\big ) - 1 \Big ) ^\frac{1}{n}, \end{aligned}$$

where $\alpha \in \mathbb {R^{+}}$ and $n \in \mathbb {R^{+}}$ are free parameters and $m=1 - \frac{1}{n}$. The retention curve of a 30/40 sand^25,59^ in this model is shown in Fig. 4.

There are many approaches to modelling hysteresis^60–63^. We want to keep the model as simple as possible, thus we adopt the simplest approach, which assumes that all the scanning curves are almost vertical line segments. Thus, if a block is transitioning between the main wetting and draining branch, it follows a straight line given by

3 $$\begin{aligned} \frac{dP}{dS} = K_{PS}, \end{aligned}$$

where $K_{PS}$ is a large constant. This enables the pressure to change rapidly while keeping the saturation almost constant. If a block undergoes wetting along a scanning line and reaches the main wetting branch, it sticks to it and further wetting proceeds along the main wetting branch. A similar principle applies in the draining mode: once a block reaches the main draining branch, it sticks to it.

Following a change in saturation, the pressure in each block is updated in this way. The pressure–saturation curve is assumed to be satisfied at all times, i.e. there is no relaxation time involved.

### Flux update

Once the pressure in each block is updated, new values of the fluxes among the blocks are calculated. The flux is modeled by the standard Darcy-Buckingham law^11^, which takes the following form:

4 $$\begin{aligned} q = \frac{\kappa }{\mu } k(S) \left( \rho g - \nabla P \right) , \end{aligned}$$

where $\kappa$ [${\text {m}}^{2}$] denotes the intrinsic permeability of the medium, $\mu$ [Pas] denotes the dynamic viscosity of the fluid, $\rho$ [${\text {kgm}}^{-3}$] denotes the density of the wetting fluid, *g* [${\text {ms}}^{-2}$] denotes the acceleration resulting from gravity, and *P* [Pa] denotes the pressure in the wetting fluid given by the retention curve. The function *k*(*S*) [-] stands for the relative permeability, i.e. the ratio of the effective permeability at a particular saturation to the intrinsic permeability. The relative permeability is usually modelled by a power law. Here we adopt the form derived in^58,64,65^:

5 $$\begin{aligned} k(S) = S^\lambda \Big [ 1 - \Big ( 1- S^{\frac{1}{m}} \Big )^m \Big ]^2, \end{aligned}$$

where $\lambda$ is a free parameter and $m=1 - \frac{1}{n}$ are the parameters of the retention curve given by equation (2). Because we are especially interested in the fingertip behaviour (which is in the imbibition mode), we always use the value *m* corresponding to the main wetting branch. Let us denote the effective permeability of the porous medium $\gamma (S) = \kappa k(S)$.

The flux update is the only step where we slightly depart from the standard implementation of the Darcy-Buckingham law. We proposed the following discrete implementation

6 $$\begin{aligned} q[(i_1,j_1) \rightarrow (i_2,j_2)] = \left\{ \begin{array}{lll} \frac{1}{\mu } \sqrt{\gamma (S(i_1,j_1)\gamma (S(i_2,j_2)} \left( \rho g - \frac{P(i_2,j_2) - P(i_1,j_1)}{dx} \right) \qquad &{} \text{ for } j_1=j_2,~ i_2=i_1+1 \\ \frac{1}{\mu } \sqrt{\gamma (S(i_1,j_1)\gamma (S(i_2,j_2)} \left( 0 - \frac{P(i_2,j_2) - P(i_1,j_1)}{dx} \right) \qquad &{} \text{ for } i_1=i_2,~ j_2=j_1+1 \\ 0 &{} \text{ otherwise } \end{array} \right. \end{aligned}$$

Thus, the lateral fluxes ($i_1=i_2$) do not include the force of gravity and the vertical fluxes do ($j_1=j_2$) include the force of gravity. Each block is assumed to have four neighbours; diagonal fluxes are not included. The geometric mean of the permeability of the neighbouring blocks is used. This is crucial because the effective permeability becomes small if at least one of the blocks is dry enough to have low permeability. This does not hold for the more typical arithmetic mean, so the use of the geometric mean proves essential for correctly capturing the behaviour of the finger tips. Further justification for using this type of averaging comes from^66^, where the authors show that the geometric mean is appropriate by means of numerical experiments in random pore networks.

By setting the fluxes among the blocks, we can update the time to $t+dt$ and proceed back to the saturation update step (1). This closes the modelling loop. Let us again stress that *dx* is not a discretizing parameter, unlike *dt*, which can be taken arbitrarily small. In practice, *dt* has to be set to be small enough to avoid numerical instability. The question of the scaling of the model with *dx* is considered in the “Discussion” section.

### Initial and boundary conditions

The two dimensional Hele-Shaw cell of a porous medium is modelled by an $N \times M$ grid of blocks. For the model to be specified fully, initial and boundary conditions must be set. The initial condition can be defined either by prescribing the initial saturation $S_{in}(i,j)$ in each block or by prescribing the initial pressure value $P_{in}(i,j)$ in each block. At the beginning, all blocks are set to start on the main wetting branch. A constant flux $q_B$ is prescribed across the top edge of each of the blocks in the top row. In experiments, a layer of very fine sand^47,54,56,57^ is usually used instead. This layer smooths out any heterogeneity in the influx, and so it simulates a constant flux across the top boundary. The lateral boundaries are assumed to be impenetrable and so zero lateral flux is prescribed there. Finally, a free discharge is set at the bottom boundary by the relation

7 $$\begin{aligned} q[(N,j) \rightarrow \text{ out}] = \frac{1}{\mu } \gamma (S) \left( \rho g + \frac{P(N,j)}{dx} \right) , \end{aligned}$$

where *N* stands for the bottom row index. Moreover, the flux from the bottom boundary is set to zero if the saturation of the respective block does not exceed a residual saturation $S_{rs}$. Otherwise, Eq. (7) is used. This implementation of initial and boundary conditions is standard and similar to models based on the Richards’ Eq.^67^.

## Results

In this section, we demonstrate the capability of the model to reproduce the experiments by Glass et al.^15,47,54–56^ and Rezanezhad et al.^57^. The experiments were concerned with the persistence of the fingers, their structure (the mobile core and immobile fringe; see below), and the dependence of the fingers on the initial saturation. Unless otherwise stated, the parameters used for the simulations are given in Table 1. The choice of the block size *dx* was inspired by the Macro Modified Invasion Percolation model developed by Glass et al.^49,50^.

**Table 1:** Parameters used for reproducing the wetting front instability experiments. The parameters for 30/40 sand were adopted from Schroth et al.^59^ and DiCarlo^25^.

Parameter	Symbol	Value
Horizontal width of the chamber	*A*	100 cm
Vertical length of the chamber	*B*	50 cm
Block size	*dx*	0.5 cm
Porosity	$\theta$	0.35
Density of water	$\rho$	1, 000 ${\text {kgm}}^{-3}$
Dynamic viscosity of water	$\mu$	$9 \times 10^{-4} \, \hbox {Pas}$
Intrinsic permeability	$\kappa$	$1.376 \times 10^{-10}$ ${\text {m}}^{2}$
Relative permeability exponent	$\lambda$	0.8
Acceleration resulting from gravity	*g*	9.81 ${\text {ms}}^{-2}$
Wetting curve parameter	$\alpha _w$	0.173 ${\text {Pa}}^{-1}$
Wetting curve parameter	$n_w$	10.00
Draining curve parameter	$\alpha _d$	0.067 ${\text {Pa}}^{-1}$
Draining curve parameter	$n_d$	13.10
Slope of scanning curves	$K_{PS}$	$10^5$ Pa
Residual saturation	$S_{rs}$	0.05
Boundary flux	$q_B$	$8 \times 10^{-5}$ ${\text {ms}}^{-1}$

**Figure 1:**
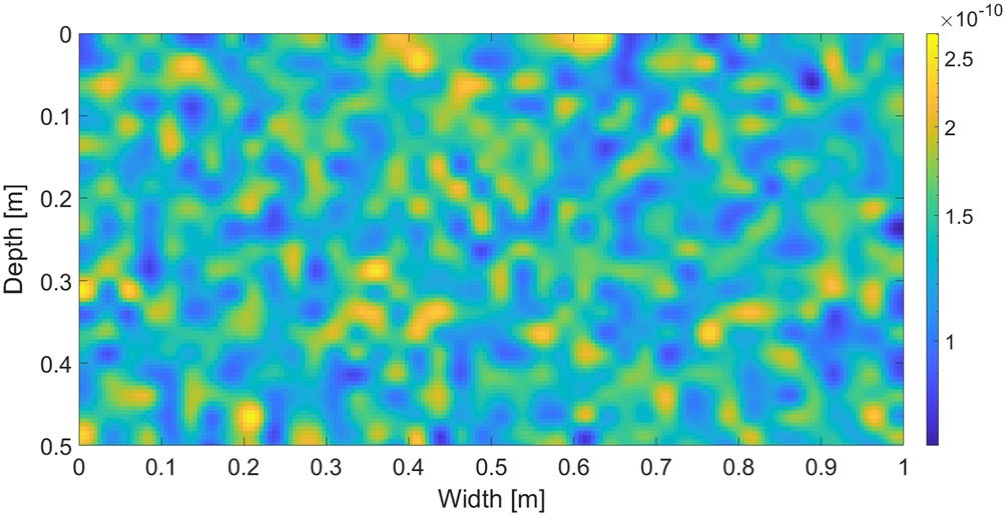
The distribution of the intrinsic permeability. Intrinsic permeability values are colour-coded according to the colour bar on the right.

The porous medium is assumed to be homogeneous, i.e. there are no preferential pathways “hard-wired” into the porous matrix structure. This, however, does not mean that it is sensible to assume that the characteristics of all the blocks are the same. Such a model would resemble an artificial medium (e.g. uniformly packed glass beads) rather than a realistic porous matrix. Thus, we introduce a narrow and spatially correlated distribution of the intrinsic permeability of the blocks. This is the only parameter that is assumed to have a distribution; otherwise the blocks are identical. The distribution satisfies $\kappa _{max} / \kappa _{min} \approx 4$ and the mean of the intrinsic permeability is approximately equal to $\kappa$. The distribution of the values of intrinsic permeability is shown in Fig. 1. A similar spatially correlated permeability field was also used in^32,33^; moreover, the authors introduce a perturbation directly into the wetting front at the beginning of the simulation.

### Finger persistence

**Figure 2:**
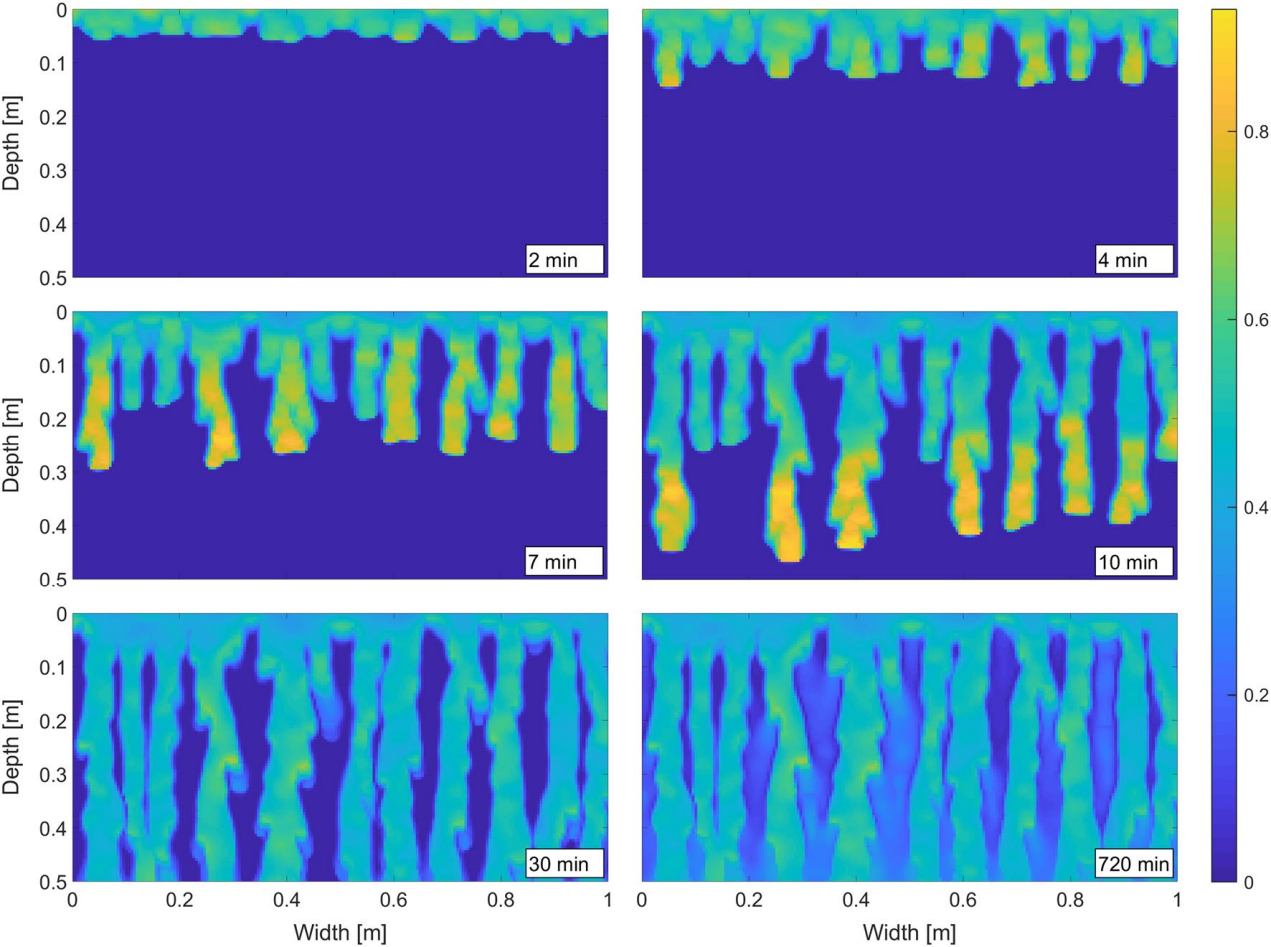
Fingering pattern at various simulation times. Constant flux over the top boundary into an almost dry isotropic porous medium. See Table 1 for the simulation parameters. For details, refer to the text. Saturation values are colour-coded according to the colour bar on the right.

First, we wish to demonstrate the instability of the wetting front—the formation of macroscopic fingers—and their persistence in time. In experiments, this fingering regime is observed for low initial saturation of the matrix^54,68^, and thus we set $S_{in}=0.01$. Figure 2 shows six snapshots of the saturation field at various simulation times. The wetting front becomes unstable and produces fingers that are very typical of an initially almost dry porous medium. Note that the flux across the top boundary is constant and no perturbation is used to initialize any instability. This is in contrast with e.g. Cueto-Felgueroso et al.^32^ or Gomez et al.^33^, who initiate fingers by a perturbation of the wetting front at the beginning of the simulation. The fingers are fully developed after approximately 10 minutes and then they proceed downward until they reach the bottom boundary. The saturation in the fingers exhibits the typical overshoot pattern—the bottom part of the finger is close to full saturation while the finger tail is much drier. The length of the oversaturated zone is approximately 17 cm, which matches well with the experiments^15^. One may observe the long persistence of the fingers (12 hours of flow is simulated), which was experimentally observed e.g. in^15,54,55,57^. Moreover, Glass et al.^15,54,55^ and DiCarlo et al.^18^ reported that after hours or days of steady infiltration, the chamber was fully wet; however, most of the flow was confined to the original finger cores and the saturation was much lower around the fingers. This is exactly what can be seen in the last snapshot in Fig. 2.

**Figure 3:**
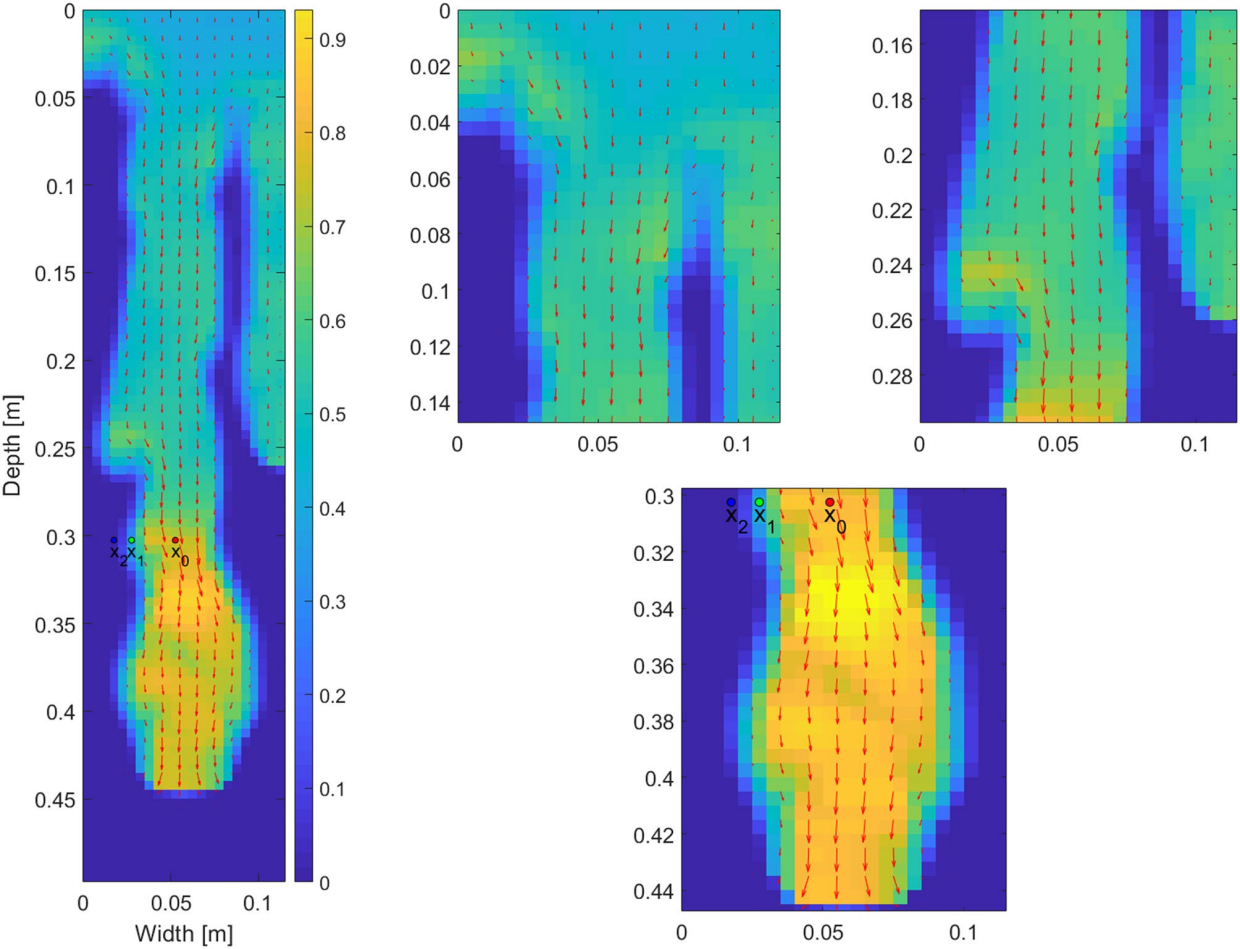
A manifestation of the mobile core and immobile fringe of a finger. Detail of the left-most finger in Fig. 2 at time 10 mins. Each arrow was produced by averaging the flux over four neighbouring blocks. Saturation values are colour-coded according to the colour bar on the right.

The persistence of fingers was explained e.g. by Glass et al.^54^ and Rezanezhad et al.^57^. Using a dye tracer, the authors showed that the flow is dominant in the centre of the fingers (the finger core) and water stagnates at the periphery (the finger fringe). Therefore, each finger is separated into a mobile core and an immobile fringe. Figure 3 shows the velocity field in the left-most finger at the time 10  minutes. For better visualization, each arrow was produced by averaging the flux vector over four neighbouring blocks. It is observed that the magnitude of the flow decreases rapidly toward the boundary of the finger and becomes negligible at the fringe.

**Figure 4:**
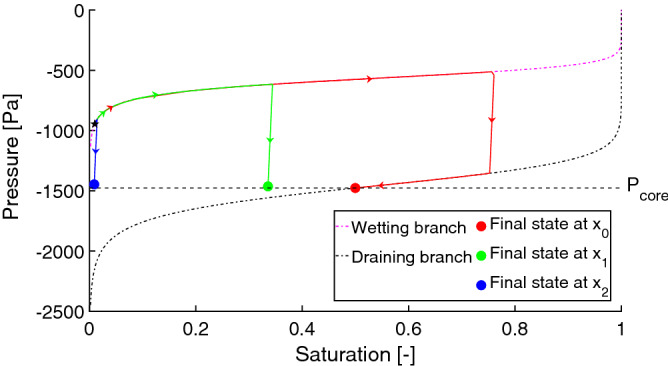
Evolution of the pressure-saturation states of three blocks $x_0$ (core), $x_1$ (between the core and the fringe), and $x_2$ (outer block of the fringe) during the first 12 minutes of the simulation. All three points started at the location denoted by a black pentagram. See the text for details.

Understanding the stability of such a flow structure is crucial and it can be explained by tracking the pressure-saturation states of the blocks on the retention curve during the passage of the finger^15,57^. Figure 4 tracks the pressure-saturation states of three points inside the finger. Point $x_0$ lies at the centre of the core (see Fig. 3), point $x_1$ lies between the core and the fringe, and point $x_2$ lies at the outer edge of the fringe. At the beginning, the porous medium was almost dry and so the pressure was negative and quite high at all three points. All three points started at the wetting branch (the top curve in Fig. 4) of the retention curve, close to its left-most point. When the finger tip was passing through $x_0$, the saturation increased quickly and the block followed the main wetting branch of the retention curve. Behind the finger tip, saturation started to decrease and, the block jumped down to the main draining branch (the almost vertical red line segment) and then moved to the left along the draining branch. During the same time, the saturation first increased at $x_1$ (the top part of the green curve) and then it decreased somewhat so that this point stayed between the main branches of the retention curve and reached almost the same pressure as the point $x_0$. Thus, the pressure difference between the points $x_0$ and $x_1$ is negligible and no flow is induced between them. This means that the structure becomes stable although the difference in saturation is large. At the point $x_2$ the evolution of pressure and saturation is similar to the point $x_1$. The finger does not expand laterally, because the pressure at $x_2$ is less negative than the pressure at $x_1$. This is due to the chosen shape of the retention curve—the pressure at the finger tail (given by the middle part of the draining curve) is always more negative than the initial pressure (given by the left-most point of the wetting curve). This is in contradiction with Rezanezhad et al.^57^ where the authors claim that the pressure at $x_2$ is much more negative than at $x_1$, and hence the finger expands slowly. Moreover, this lateral finger expansion was also experimentally observed^15,54,55^.

**Figure 5:**
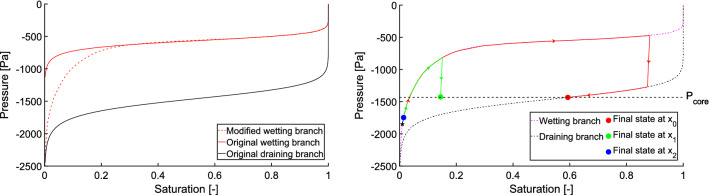
Lateral expansion of the fingers. Left panel: The modified retention curve of 30/40 sand. Original van Genuchten wetting curve (full red line) and its modification by a cubic spline (dotted red line). The draining branch (full black line) was not modified. Right panel: Evolution of the pressure-saturation states of three blocks $x_0$ (core), $x_1$ (between the core and the fringe), and $x_2$ (outer block of the fringe) during the first 20 minutes of the simulation. All three points started at the location denoted by a black pentagram. See the text for details.

We suggest that the model presented here may help to explain the lateral expansion of the fingers. In the previous paragraph, we explained that our choice of the retention curve led to fingers that do not expand laterally. However, a modification of the retention curve shape allows for lateral expansion. Let us assume a retention curve with a larger gradient in the left part (i.e. steeper dependence of pressure on saturation in a dry medium). Since it is difficult to obtain such a retention curve by the van Genuchten model^58^, we modified the wetting branch by means of a cubic spline. The left panel of Fig. 5 shows the modified wetting branch (dotted red line) compared to the original van Genuchten model (full red line). The main draining branch (full black line) was not modified. This modification allows blocks on the wetting branch at initial saturation to attain more negative pressure compared to the finger tail.

Let us repeat the simulation shown in Fig. 2 with this modified retention curve. As a result of the high gradients of the retention curve for low saturations, it was necessary to increase the parameter $\lambda$ in the relative permeability Eq. (5). Here, we set $\lambda =1.5$. All the remaining parameters of the simulation remained the same. The character of the flow is almost identical to the model using the van Genuchten retention curve. This shows that the fingering regime is very robust—a rather dramatic change in the retention curve has little effect on the finger formation process; see Supplementary Video 2. However, in this case, gradual lateral expansion of the fingers may be observed (the difference is already visible after 20 minutes of simulation). To understand the difference between the original (van Genuchten) and modified retention curve, we again demonstrate the evolution of the pressure-saturation hydraulic states for three locations, $x_0,$ $x_1,$ and $x_2$ in right panel of Fig. 5. Analogously to Fig. 4, the pressure difference between the points $x_0$ and $x_1$ was negligible after the first 20 minutes of the simulation and so the flux between the points was also negligible. The saturation at the point $x_2$ increased slightly from its initial value and so the pressure moved along the main wetting branch. But because of the modified retention curve, the pressure at $x_2$ was still lower than the pressure at the fringe, and so there was outward lateral flux from the fringe of the finger. This is in agreement with the observation of Rezanezhad et al.^57^ and explains the slow lateral expansion of the fingers in Supplementary Video 2.

### The effect of initial saturation

Let us now examine the effect of the initial saturation $S_{in}$ on the evolution of the flow patterns. In experiments, this dependence is observed to exhibit very interesting qualitative and quantitative features^27,69^. Most interestingly, as the initial saturation increases from dry to fully saturated, the flow pattern changes qualitatively from the fingering regime (a complex network of preferential pathways, each with the saturation overshoot effect) to a diffusion-like wave water front travelling uniformly (without any overshoot). Moreover, the transition from the fingering regime to the diffusion regime is not monotonic. With increasing initial saturation, the fingers first become faster and narrower, but then they become slower and wider again before disappearing completely into the diffusion-like regime. The model presented here correctly captures both the qualitative and quantitative aspects of this wonderful transition. We again wish to stress that the model does not introduce any artificial or non-measurable parameters to do so.

**Figure 6:**
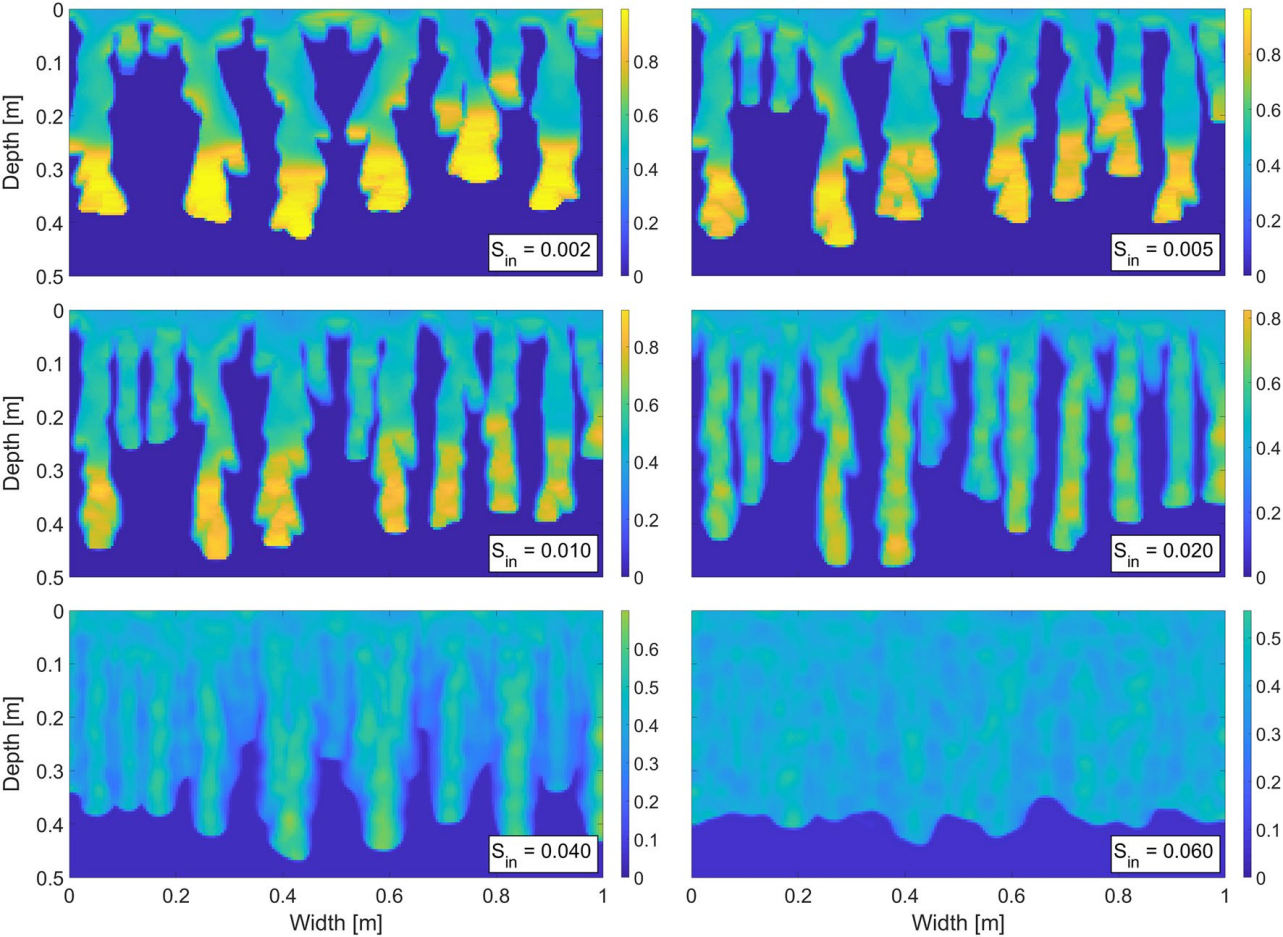
Snapshot of the saturation field at 10 mins for six different values of the initial saturation. See the Supplementary Information for full videos. Saturation values are colour-coded according to the colour bar on the right.

Figure 6 shows a snapshot of the saturation field at 10 mins for six different values of the initial saturation. See the Supplementary Information for full videos. The model seems to be in complete agreement with the observed transition from the fingering regime to the diffusion-like regime.

**Table 2:** Parameters used for reproducing the flow across layers of porous media with different characteristics. Parameters not included in this table were the same as in Table 1.

Parameter	Symbol	Value
Intrinsic permeability (homogeneous):	$\kappa$	$1.376 \times 10^{-10}$ ${\text {m}}^{2}$
Retention curve parameters (homogeneous):	$\alpha _w$	0.173 ${\text {Pa}}^{-1}$
	$n_w$	10.00
	$\alpha _d$	0.067 ${\text {Pa}}^{-1}$
	$n_d$	13.10
Intrinsic permeability (heterogeneous):	$\kappa$	$4.587 \times 10^{-11}$ ${\text {m}}^{2}$
Retention curve parameters (heterogeneous):	$\alpha _w$	0.1471 ${\text {Pa}}^{-1}$
	$n_w$	7.00
	$\alpha _d$	0.0623 ${\text {Pa}}^{-1}$
	$n_d$	9.17
Width of the chamber	*A*	50 cm
Depth of the chamber	*B*	150 cm

### Flow across layers of porous media with different characteristics

The experiments of Rezanezhad et al.^57^ provide us with an opportunity to test the model in the situation when a layered porous medium is subject to a constant influx of water across the top boundary. Rezanezhad et al.^57^ use a Hele-Shaw cell ($160 \times 60 \times 0.3 \, \hbox {cm}$) filled with four layers of porous material with different characteristics. The top layer ($5~ \, \hbox {cm}$ deep) was composed of fine sand with a grain size diameter between 0.063 mm and 0.25 mm, the second and the bottom layers (both $50~\text {cm}$ deep) consisted of sand with a grain size $0.63-1.25$ mm (called homogeneous sand), and these two layers were separated by a layer of horizontally arranged sand (also $50~\text {cm}$ deep) with a grain size between 0.25 mm and 1.25 mm (called heterogeneous sand).

Thus, the separating layer (heterogeneous sand) has smaller grains on average, and therefore its retention curve should be steeper compared to the homogeneous layers. The saturated hydraulic conductivity of the heterogeneous layer was approximately three times lower than that of the homogeneous layers. For details, see Rezanezhad et al.^57^.

**Figure 7:**
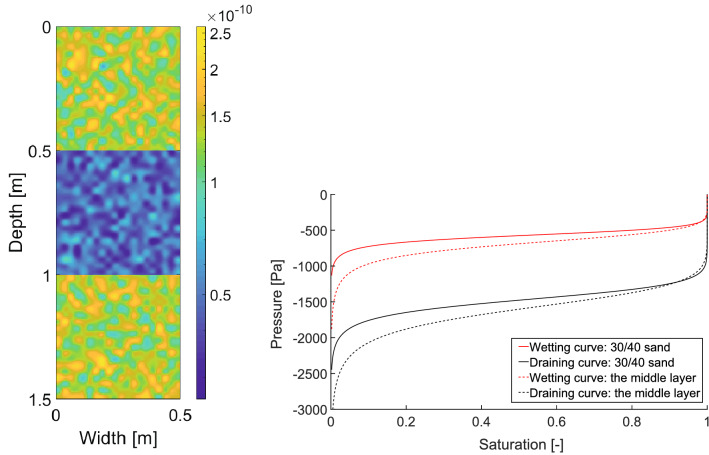
Reproducing the flow across layers of porous media with different characteristics. Left panel: The distribution of the intrinsic permeability. The values of intrinsic permeability are colour-coded according to the colour bar on the right. Right panel: The retention curves of the respective layers.

In the experiment, the top $5~\text {cm}$ layer served as a “randomizer” to smooth out any heterogeneity in the influx. In the model, it is not a problem to keep the influx exactly homogeneous, and thus we do not simulate the top layer. For the simulation of the three $50~\text {cm}$ layers beneath, we used the parameters shown in Table 2. All the other parameters of the simulation were identical to the previous two runs of the model. Both the retention curves and the spatial distribution of intrinsic permeability are shown in Fig. 7. The horizontal arrangement of the heterogeneous layer was ignored in the simulation.

**Figure 8:**
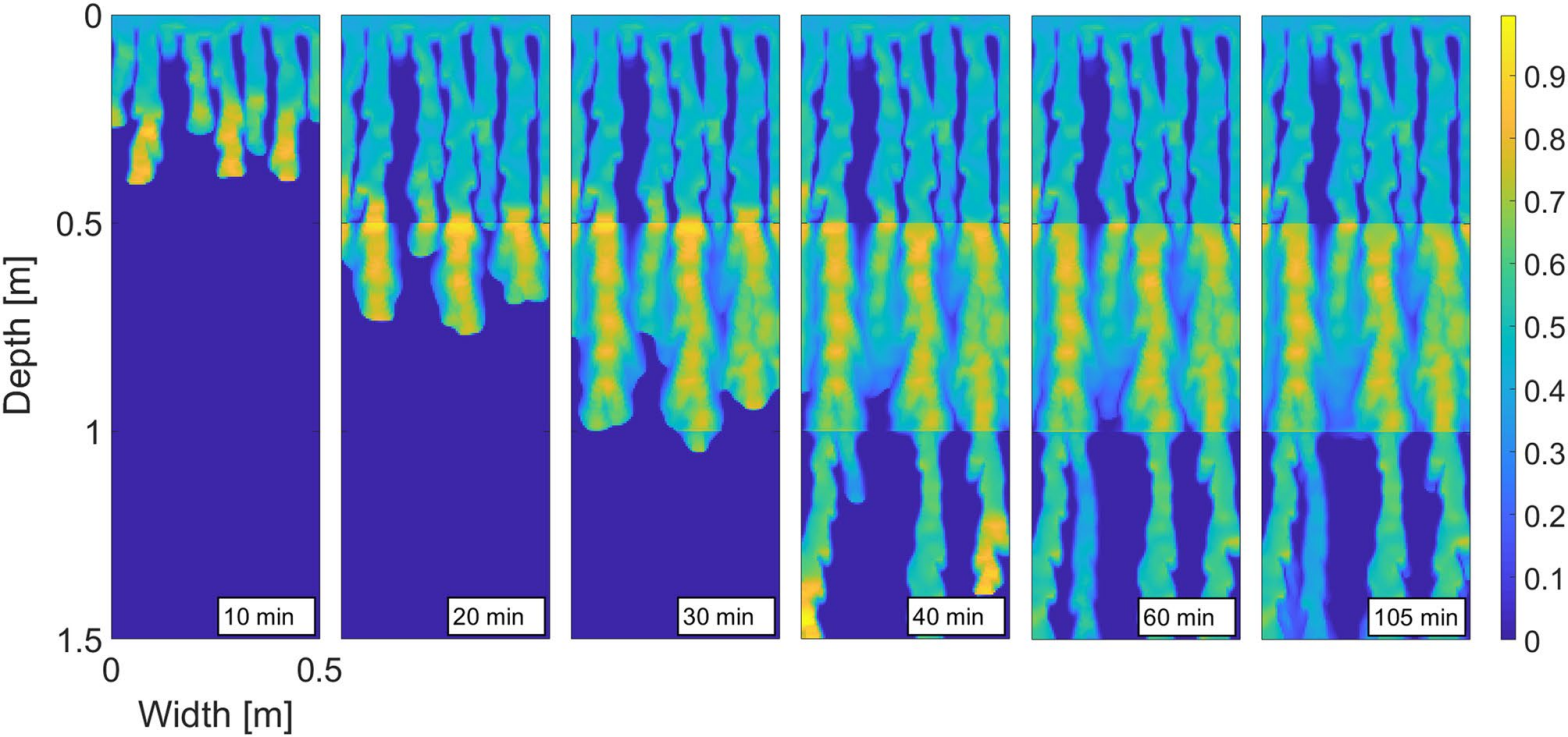
Simulated flow across layers of porous media with different characteristics. Snapshots of the saturation field for several successive time points. Saturation values are colour-coded according to the colour bar on the right.

Rezanezhad et al.^57^ showed that stronger capillary forces in the heterogeneous layer (because of the smaller grains) were sufficient to disturb the finger flow. However, the fingering pattern re-appeared immediately in the bottom layer (see Fig. 5 in Rezanezhad et al.^57^ or the supplementary video therein). We reproduce this behaviour in Fig. 8, which shows six successive snapshots of the saturation field produced by the model. Observe that the second layer wiped out the typical fingering pattern and a more diffusive regime appeared. When the fingers re-appeared in the bottom layer, there were fewer of them compared to the top layer. This is also consistent with the experimental findings^57^. If we introduced stronger horizontal spatial correlation of the intrinsic permeability in the middle layer (corresponding to the horizontal arrangement), the flow pattern would be disturbed even more. Here, we only wanted to show that this effect can be reproduced by a small change in the retention curve.

## Discussion

The difficulty of modelling unsaturated porous media flow can be best understood by studying the effect of the initial saturation of the matrix on the flow pattern. As the initial saturation increases, the flow pattern changes qualitatively from the fingering regime to a diffusion-like regime. During this transition, the saturation overshoot gradually disappears and the finger width depends non-monotonically on the initial saturation (see the Results section). Macro Modified Invasion Percolation models^48,49^ are moderately successful in reproducing the finger-like regime; however, the diffusion-like behaviour is beyond their capacity because there is no way to recover continuous saturation levels and physically meaningful time. At the other end, the Richards’ equation is known to reproduce the diffusion-like behaviour but the fingering regime is beyond its power as a result of impossibility of its producing the saturation overshoot^22^. Other models capture some features of the flow in the diffusion like regime or in the fingering regime, but there is usually a price—new parameters that are impossible to measure^28,32,35^, and/or artificial boundary conditions^34,70^ and/or disagreement with the experiments in other flow regimes except for the one that the model was tuned to^71^. The model presented here captures both the qualitative and quantitative aspects of unsaturated porous media flow both in the fingering regime and in the diffusion regime.

To support this rather bold claim, it is crucial to understand the role of the block size *dx*. If we let $dx \rightarrow 0$ (obviously forcing $dt \rightarrow 0,$ accordingly), and kept other parameters constant, we would yield the Richards’ equation. This is independent of the geometric mean used in the model because all the means (arithmetic, geometric, and harmonic) converge to the same value when both the averaged numbers converge to a single value. This limit, however, is wrong for the following reasons. Even as $dx \rightarrow 0,$ each block retains the nature of a porous medium in the sense that it is characterized by its retention curve. If a block becomes smaller, the variability of the geometry of its pore-space decreases, and consequently its retention curve becomes simpler. If we imagine a block so small that it contains only a single pore, its retention curve becomes completely flat. For a single pore, the transition from zero to unit saturation happens at a constant pressure (the water-entry pressure) and the transition from unit to zero saturation also happens at constant pressure (the air-entry pressure). Thus, in the limit $dx \rightarrow 0,$ the retention curve of each block collapses to two parallel horizontal lines, one for imbibition and the other one for drainage. Such a retention curve is inadmissible in the context of the RE and so the semi-continuum model does not reduce to the RE in the limit $dx \rightarrow 0$. Thus, the presented semi-continuum model is not a numerical scheme to solve the RE and it does not reduce to the RE even in the limit $dx \rightarrow 0.$ The limiting process will be thoroughly addressed in a subsequent article, however an example of the block size scaling is provided in the Supplementary Information.

It is well known that forward time discretization (see Eq. 1) may cause instability of the numerical simulations which usually manifest themselves as spurious oscillations in the finger tip. One may suspect that these spurious oscillations cause the overshoot behavior of the model. To this end, we implemented a backward time discretization of the semi-continuum model. We compared the explicit and implicit discretizations in a one dimensional setting parameters identical to those used in the two dimensional simulations. We show that the overshoot behavior is independent on the discretization scheme used. This comparison, together with a time step analysis for both the numerical schemes is presented in the Supplementary Information.

Experiments show that fingers can merge or bifurcate^15,23,57^, which is well reproduced by the semi-continuum model. A close look at the simulations reveals two different scenarios of finger conjunction: the fingers either merge (usually by coalescence of their tips) or they converge and flow side by side without merging. In the first case, the saturation at the finger tip is high enough to break through the immobile fringes. Fingers also merge if the hydraulic conductance below the finger tip is low enough to force lateral expansion of the finger. On the other hand, sometimes two fingers converge without merging. This happened e.g. to the second and the third fingers from the left in Fig. 2 (see Supplementary Fig. 3 for more details). In this case, neither of the fingers was able to penetrate the fringe of the other. See a more detailed discussion in the Supplementary Information.

The hysteresis of the retention curve plays an important role in the finger profile build-up. It is known that all points where saturation overshoot occurs are located on a scanning curve (i.e. between the two main branches of the retention curve, see Fig. 10 in^57^). Thus, by decreasing the length of the scanning curves (i.e. decreasing the “gap” between the two main branches), the length of the over-saturation zone of the finger also decreases. This is consistent with our simulations, see Supplementary Fig. 5 for more details.

## Conclusion

In this article, we propose combining the virtues of continuum and discrete modelling of fluid flow through an unsaturated porous medium. Continuum models work well for diffusion-like regimes but fail to capture preferential flow (finger flow) and its characteristics, especially the saturation overshoot phenomenon. Invasion percolation models and their modifications, on the other hand, capture some features of finger flow but usually describe saturation as a binary variable thus missing a large part of the physical reality. The proposed model describes pressure and saturation as fields that are continuous in time but piecewise constant in space. This compromise between continuous and discrete modelling is able to reproduce (1) gravity-induced preferential flow with a spatially rich system of rivulets (fingers) characterized by saturation overshoot, (2) diffusion-like flow with a monotonic saturation profile, (3) the transition between the two. The model helps to explain the formation of the preferential pathways, their persistence and structure (the core and fringe of the fingers), the effect of the initial saturation of the matrix, and the saturation overshoot phenomenon. There remains an open question of the limit of the model as $dx \rightarrow 0$ which will be addressed in a subsequent paper. We conjecture that the semi-continuum approach may also be applicable outside the scope of porous media flow modelling.

## Supplementary Information

Supplementary Information.

Supplementary Video 1.

Supplementary Video 2.

Supplementary Video 3.

Supplementary Video 4.

Supplementary Video 5.

Supplementary Video 6.

Supplementary Video 7.

Supplementary Video 8.

Supplementary Video 9.

Supplementary Video 10.

## Data Availability

No experimental data were generated or analysed during the current study. The code that produced all the simulations is available in MatLab upon request at corresponding author.
