# Supplementary material for: A two dimensional semi-continuum model to explain wetting front instability in porous media
Source: Sci Rep. 2021 Feb 5;11:3223. doi: 10.1038/s41598-021-82317-x (PMC7864934; doi:10.1038/s41598-021-82317-x)
Supplement: Supplementary file 1 — Supplementary Information. [file 41598_2021_82317_MOESM1_ESM.pdf]

A two dimensional semi-continuum model to explain wetting front instability in porous media

Jakub Kmec[1], **Tomáš Fürst**[2], **Rostislav Vodák**[2], **and Miloslav Šír**[3,*]

[1]Palacky University in Olomouc, Joint laboratory of optics, Faculty of Science, Olomouc, 771 46, Czech Republic
[2]Palacky University in Olomouc, Dpt. Mathematical analysis and applications of mathematics, Faculty of Science, Olomouc, 771 46, Czech Republic
[3]University of South Bohemia, Institute of Aquaculture and Protection of Waters, Faculty of Fisheries and Protection of Waters, České Budějovice, 370 05, Czech Republic
[*]milo_sir@yahoo.com

Supplementary Information

Supplementary videos of simulations

In this section, complete videos are provided for each of simulations presented in the main article. The MatLab code used to generate the videos is available at JK upon request. A brief description of the Supplementary videos follows:

- *Supplementary Video* 1.*avi*: Simulation of the first 20 minutes of the evolution of the saturation field, $S_{in} = 0.010$ (see subsection “Finger persistence” in the article body).
- *Supplementary Video* 2.*avi*: Simulation of the first 20 minutes of the evolution of the saturation field for the modified retention curve, $S_{in} = 0.010$ (see subsection “Finger persistence” in the article body).
- *Supplementary Video* 3.*avi*: Simulation of the first 10 minutes of the evolution of the saturation field, $S_{in} = 0.002$ (see subsection “The effect of initial saturation” in the article body).
- *Supplementary Video* 4.*avi*: Simulation of the first 10 minutes of the evolution of the saturation field, $S_{in} = 0.005$. (see subsection “The effect of initial saturation” in the article body).
- *Supplementary Video* 5.*avi*: Simulation of the first 10 minutes of the evolution of the saturation field, $S_{in} = 0.020$. (see subsection “The effect of initial saturation” in the article body).
- *Supplementary Video* 6.*avi*: Simulation of the first 10 minutes of the evolution of the saturation field, $S_{in} = 0.030$. (see subsection “The effect of initial saturation” in the article body).
- *Supplementary Video* 7.*avi*: Simulation of the first 10 minutes of the evolution of the saturation field, $S_{in} = 0.040$. (see subsection “The effect of initial saturation” in the article body).
- *Supplementary Video* 8.*avi*: Simulation of the first 10 minutes of the evolution of the saturation field, $S_{in} = 0.050$. (see subsection “The effect of initial saturation” in the article body).
- *Supplementary Video* 9.*avi*: Simulation of the first 10 minutes of the evolution of the saturation field, $S_{in} = 0.060$. (see subsection “The effect of initial saturation” in the article body).
- *Supplementary Video* 10.*avi*: Simulation of the first 60 minutes of the evolution of the saturation field for flow across layers of porous media with different characteristics, $S_{in} = 0.010$, (see subsection “Flow across layers of porous media with different characteristics” in the article body).

Analysis of numerical simulations

It is well known that forward time discretization (see equation (1) in the article) may cause instability of the numerical simulations which usually manifest themselves as spurious oscillations in the finger tip. One may suspect that these spurious oscillations cause the overshoot behavior of the model. To this end, we implemented a backward time discretization of the semi-continuum model. We compared the explicit and implicit discretizations in a one dimensional setting parameters identical to those used in the two dimensional simulations. Supplementary Fig. 1 (left panel) shows saturation profiles for various values of initial saturation of the matrix. The simulations were performed both by the explicit and implicit numerical schemes. The results are so similar that difference is not visible in the left panel. The right panel of Supplementary Fig. 1 shows the L^2-norm of the difference between the two solutions for various values of initial saturation. The difference is negligible and it can be concluded that the choice of time discretization is irrelevant, provided the time step in the explicit scheme is small enough.

Another way to show the numerical stability of the simulations is a time step analysis. The recommended procedure is to repeat the simulation with half the original time step and compare the results. This should be repeated until the difference between two consecutive solutions is sufficient small. Supplementary Fig. 2 shows the L^2-norm of the difference between each pair of consecutive solutions for dt between 1.25×10^{-5} s and 8×10^{-4} s. The initial saturation of the matrix is set to $S_{in} = 0.01$. Each dot in the graph corresponds to the difference between the solutions for dt given by the $x-$axis and dt twice as large. The backward time discretization scheme did not converge for $dt = 8 \times 10^{-4}$ s. It can be observed that both the numerical schemes are stable for dt sufficiently small.

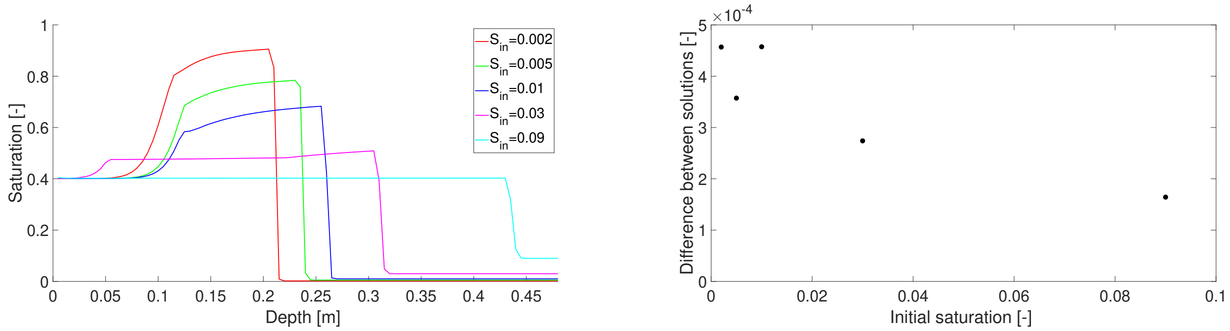

Figure 1. A comparison between a forward and backward time discretization schemes. Left panel: Saturation profiles for various values of initial saturation. No difference between both the schemes can be observed in this type of plot. Right panel: The L^2-norm of the difference between the two solutions for various values of initial saturation. Both the numerical schemes yield almost identical results.

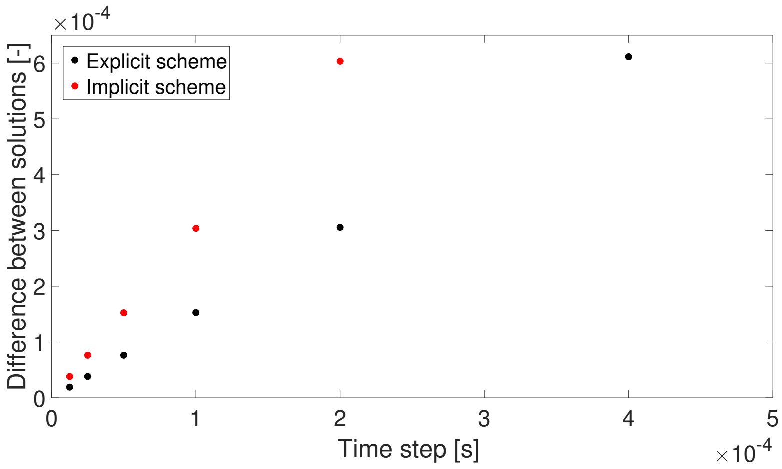

Figure 2. A time step analysis for the forward and backward time discretization schemes for $S_{in} = 0.01$. The L^2-norm of the difference between two consecutive solutions is calculated for various values of time step dt. Each dot in the graph corresponds to the difference between the solutions calculated for dt given by the $x-$axis and dt twice as large.

Fingers merging

Experiments show that fingers can merge or bifurcate[1–3]. A close look at the simulations (see the main article or videos in the Supplementary Information) reveals two different scenarios of finger conjunction: the fingers either merge (usually by coalescence of their tips) or they converge and flow side by side without merging. In the first case, the saturation at the finger tip is high enough to break through the immobile fringes. Fingers also merge if the hydraulic conductance below the finger tip is low enough to force lateral expansion of the finger. On the other hand, sometimes two fingers converge without merging. This happened e.g. to the second and the third fingers from the left in Fig. 2. In this case, neither of the fingers was able to penetrate the fringe of the other. Let us also note, that the fingers finally merged at around 16 minutes (see *Supplementary Video* 1*.avi* in the Supplementary Information). To understand this phenomenon more thoroughly, see Supplementary Fig. 3.

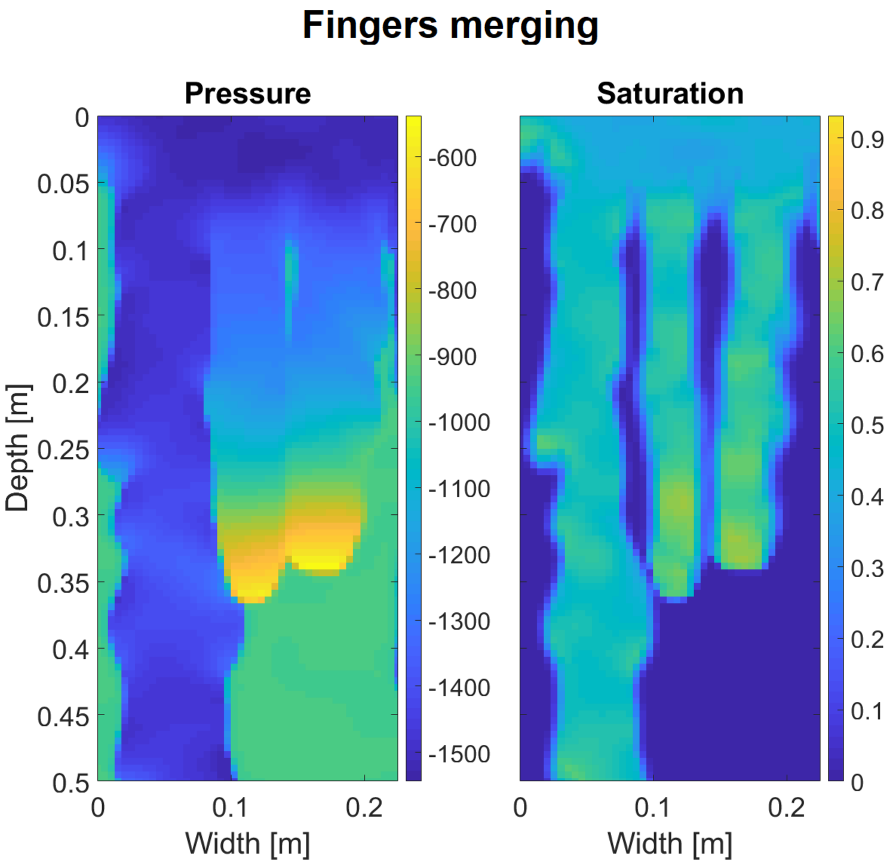

Figure 3. A snapshot of the pressure [Pa] and saturation [−] fields 13 minutes after the start of the simulation. The second and third fingers did not merge because the gradient between the fingers was equal to zero (as a result of the equality of pressure at the fringes) and thus the flux between them was negligible. The situation was different for the first and second fingers, as the slower finger collided with the tail of the faster finger. In this case, the gradient was high, but still not sufficient to break through the immobile parts of both fingers. Neither of the effects – merging of the fingers and side-by-side flow – is affected by the modification of the retention curve described above. Pressure and saturation values are colour-coded according to the colour bar on the right for each snapshot.

The limit $dx \to 0$: block size scaling

If we let $dx \to 0$ (obviously forcing $dt \to 0$, accordingly), and kept other parameters constant, we would yield the Richards' equation. This is independent of the geometric mean used in the model because all the means (arithmetic, geometric, and harmonic) converge to the same value when both the averaged numbers converge to a single value. This limit, however, is wrong for the following reasons. Even as $dx \to 0$, each block retains the nature of a porous medium in the sense that it is characterized by its retention curve. If a block becomes smaller, the variability of the geometry of its pore-space decreases, and consequently its retention curve becomes simpler. If we imagine a block so small that it contains only a single pore, its retention curve becomes completely flat. For a single pore, the transition from zero to unit saturation happens at a constant pressure (the water-entry pressure) and the transition from unit to zero saturation also happens at constant pressure (the air-entry pressure). Thus, in the limit $dx \to 0$, the retention curve of each block collapses to two parallel horizontal lines, one for imbibition and the other one for drainage. Such a retention curve is inadmissible in the context of the RE and so the semi-continuum model does not reduce to the RE in the limit $dx \to 0$. Thus, the presented semi-continuum model is not a numerical scheme to solve the RE and it does not reduce to RE even in the limit $dx \to 0$. The limiting process will be thoroughly addressed in a subsequent article, however an example of the block size scaling is provided here. The left panel of Supplementary Fig. 4 shows the scaling of the retention curve for different values of the block size dx. The right panel shows the simulated saturation profiles of the fingers computed for different values of the block size dx with the retention curve scaled according to the left panel. All the simulations are run for the initial saturation $S_{in} = 0.01$.

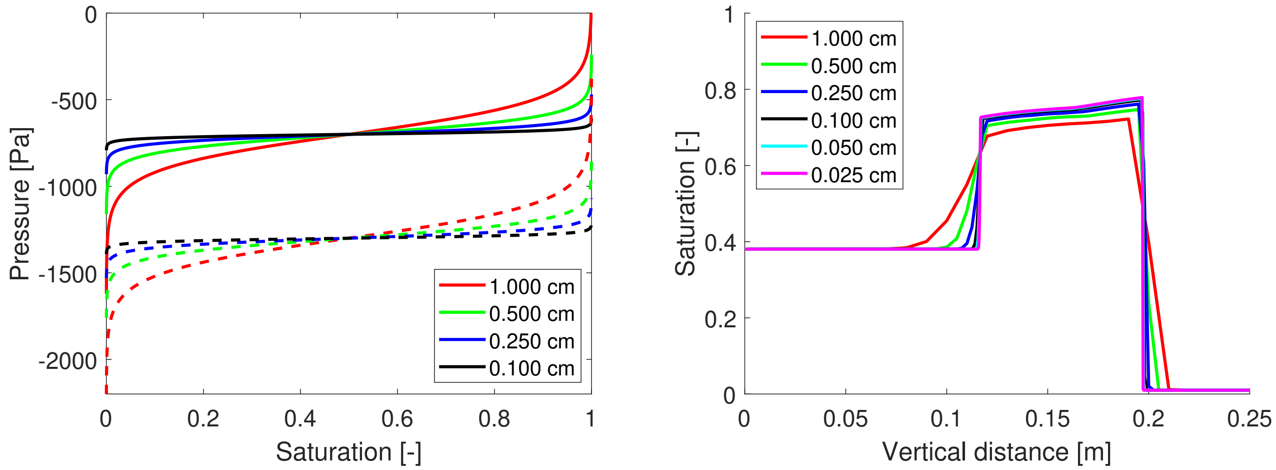

Figure 4. Left panel: The scaling of the retention curve as the block size $dx \to 0$. The solid line denotes the main wetting branch and the dashed line denotes the main draining branch. Right panel: Convergence of the simulated moisture profile of the finger at $t = 10$ minutes for $dx \to 0$ when the correct scaling of the retention curve (shown in the left panel) in used. All simulations were performed for the initial saturation $S_{in} = 0.01$. The simulated moisture profile converges and retains the overshoot pattern even as $dx \to 0$.

The effect of capillary pressure hysteresis

The hysteresis of the retention curve plays an important role in the finger profile build-up. Here, we show the effect of changing the length of the scanning curve, i.e. varying the "gap" between the two main branches of the retention curve. Supplementary Fig. 5 shows four snapshots of the solution for four different choices of the length of the scanning curve. The initial saturation was kept at $S_{in} = 0.01$ for all the simulations. The distance between the two main branches of the experimentally measured retention curve of $30/40$ sand is approximately 900 Pa at the midpoint $S = 0.5$. The main wetting branch was kept constant for all simulations and the main draining branch was made to gradually approach the main wetting branch so that the difference between the two main branches at midpoint becomes 900, 600, 200, and 0 Pa respectively. For the limiting case without hysteresis (i.e. zero length of the scanning curves), we simply used the same parameters for both the main branches. It is known that all points where saturation overshoot occurs are located on a scanning curve (i.e. between the two main branches of the retention curve, see Fig. 10 in[3]). Thus, by decreasing the length of the scanning curves (i.e. decreasing the "gap" between the two main branches), the length of the over-saturation zone of the finger also decreases. This is consistent with our simulations. Let us notice that the saturation in the bottom-most block of the fingertip is given only by the main wetting branch of the retention curve. The saturation there increases to a certain value which is higher than the saturation in the finger tail. Thus, for the zero hysteresis case, the oversaturated zone of the finger consists of a single block.

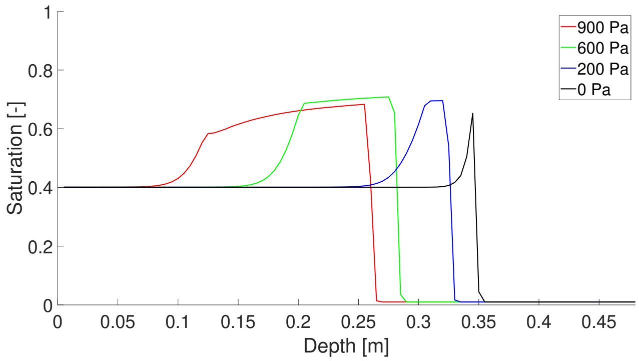

Figure 5. The effect of changing the length of the scanning curve for $S_{in} = 0.01$. The simulation for the experimentally measured retention curve of $30/40$ sand is plotted in red. The legend indicates the difference between the two main branches of the retention curve at the midpoint $S = 0.5$. The simulation results are almost identical for explicit and implicit numerical schemes.

References

1. Glass, R. J., Parlange, J.-Y. & Steenhuis, T. S. Mechanism for finger persistence in homogenous unsaturated, porous media: Theory and verification. *Soil Sci.* **148(1)**, 60–70 (1989).
2. Glass, R. J., Parlange, J.-Y. & Steenhuis, T. S. Wetting front instability. 2. experimental determination of relationships between system parameters and two-dimensional unstable flow field behavior in initially dry porous media. *Water Resour. Res.* **25(6)**, 1195–1207 (1989).
3. Rezanezhad, F., Vogel, H.-J. & Roth, K. Experimental study of fingered flow through initially dry sand. *Hydrol. Earth Syst. Sci. Discuss.* **3(4)**, 2595–2620 (2006).
